# APOBEC3 in papillomavirus restriction, evolution and cancer progression

**DOI:** 10.18632/oncotarget.6324

**Published:** 2015-11-13

**Authors:** Cody J. Warren, Dohun Pyeon

**Affiliations:** Department of Immunology and Microbiology, Division of Infectious Diseases, Department of Medicine, University of Colorado School of Medicine, Aurora, Colorado, USA

**Keywords:** APOBEC3, virus restriction, cancer mutagenesis, virus evolution, papillomavirus

The APOBEC3 family of cytidine deaminases plays critical roles in host innate immunity. These enzymes function as viral DNA mutators capable of restricting an array of virus infections [1]. Given the potent antiviral function of APOBEC3s, strategies to enhance their activity have been suggested as a novel class of antiviral agents. However, recent cancer genome studies have highlighted concerns regarding such an approach. Substantial off-target effects of APOBEC3A (A3A) and APOBEC3B (A3B) activity on host genomic DNA has been noted as a mechanistic source for somatic mutations in various human cancers [2, 3]. Despite the connection between A3A/A3B and cancer mutagenesis, it remains unclear what triggers and promotes off-target APOBEC3 activity during cancer progression.

Human papillomavirus (HPV)-associated cancers, including cervical cancer and a subset of head and neck cancers, are enriched in APOBEC3-type mutation signatures [2]. To determine key pathways involved in HPV-associated cancer progression, we performed a global gene expression analysis of cervical tissue samples in different disease stages (normal, early and late premalignant lesions and cancer) [4]. We found that A3A and A3B expression was significantly increased in low- and high-grade lesions when compared with normal tissue. Furthermore, elevated expression of A3A and A3B was observed in keratinocytes that harbor high-risk HPV genomes [5]. Collectively, these results provide a link between persistent viral infection and increased A3A and A3B expression levels. Our studies, combined with observations from other groups [Reviewed in 6], lead us to speculate that somatic mutations driven by A3A and A3B deamination may accumulate early in HPV-infected epithelial cells and continue for decades during cancer progression.

HPV is one of the most common sexually transmitted pathogens. Nevertheless, the vast majority of HPV infections are self-limiting, suggesting that host immunity is efficient at eliminating the virus. We recently found that A3A, but not A3B, inhibits HPV infection in a deaminase dependent manner [5]. It is intriguing that A3A upregulation occurs throughout disease progression, likely in an attempt by the innate immune system to limit persistent viral infection. Increased APOBEC3 levels may serve as a double-edged sword by both limiting virus activity and facilitating off-target somatic mutations that may lead to cancer mutagenesis.

Elevated A3A expression in HPV-positive cells may limit viral fitness. Thus, HPV must counteract A3A to cope with its restrictive effects. The HIV-1 viral infectivity factor (Vif), one of six accessory proteins, blocks the antiviral activity of APOBEC3G (A3G) by promoting its ubiquitination and proteasome-dependent degradation [1]. Our analysis revealed that the HPV oncogenes E6 and E7, which also regulate ubiquitin-mediated protein degradation, did not negatively alter A3A protein levels (unpublished data). This implies that HPV likely employs alternative strategies to evade host restriction by APOBEC3.

APOBEC3s deaminate cytidines with a preferred dinucleotide context. A3G preferentially targets CC dinucleotides, while the remaining family members (APOBEC3A/B/C/D/F/H) target TC dinucleotides [1]. Given the specific anti-HPV effects of A3A, we hypothesized that HPV genomes are depleted in TC dinucleotides to limit the restrictive effects of A3A. To address this hypothesis, we analyzed the relative abundance of all dinucleotide sequences in the genomes of 274 papillomaviruses documented in the Papillomavirus Episteme database (PAVE; http://pave.niaid.nih.gov). We revealed that TC dinucleotides are highly depleted in papillomavirus genomes. The extent of the depletion is dependent on the tropism of the virus, as mucosotropic viruses of the *Alphapapillomavirus* genus exhibit the greatest degree of TC depletion. Interestingly, mucosal tissues displayed significantly higher expression of various APOBEC3 family members compared to cutaneous skin [7]. The high levels of APOBEC3s in mucosal epithelium are likely due to epithelial sites being primary barriers to virus infection. Hence, the innate immune response, including restriction by APOBEC3, is more likely to be primed at these sites.

**Figure 1:**
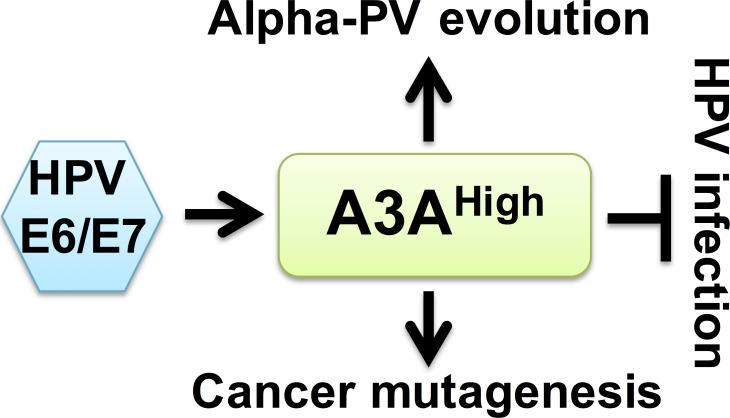
Potential link between HPV restriction, cancer progression and viral genome evolution A3A is induced to restrict HPV infection. To avoid the restrictive effects of A3A, mucosotropic HPVs have evolved with reduced APOBEC3A target sites in their genomes. During periods of viral persistence, high levels of A3A may induce somatic mutations in the host genome which promote cancer mutagenesis.

Our studies suggest a model in which the HPV oncoproteins induce A3A and A3B expression during virus persistence. If APOBEC3 activity is sufficiently high for virus restriction, HPV infection is inhibited and viral genomes are eliminated. To avoid the antiviral effects of APOBEC3, HPVs may have evolved to limit the number of TC dinucleotides in their genomes. Surviving viral genomes continue to induce A3A and A3B expression throughout disease progression, which may lead to off-target effects on the host genome that fuel oncogenic mutations (Figure 1).

Recent findings raise important questions: What additional factors and mechanisms do lead to deregulation of APOBEC3s? If cancer is considered as an evolving clonal disease, what impact does A3A and/or A3B have during this process? Can APOBEC3 activity serve as a prognostic indicator for risk of cancer progression? Further studies are necessary to understand these dynamic host-pathogen interactions to identify new ways to slow or stop disease progression.
